# Draft Genome Sequence of Saccharomyces cerevisiae DJJ01, Isolated from Dojoji Temple in Gobo, Wakayama, Japan

**DOI:** 10.1128/mra.00113-22

**Published:** 2022-07-11

**Authors:** Shinnosuke Okuhama, Kaoru Nakasone, Kazuki Yamanaka, Chiho Miyazaki, Tsumugi Nakamoto, Yuki Nakashima, Masataka Kusube

**Affiliations:** a Department of Ecosystem Engineering, Applied Engineering Faculty, National Institute of Technology, Wakayama College, Gobo, Wakayama, Japan; b Graduate School of Science, Technology, and Innovation, Kobe University, Kobe, Hyogo, Japan; c Department of Biotechnology and Chemistry, Kinki University, Higashihiroshima, Hiroshima, Japan; d Department of Materials Science, National Institute of Technology, Wakayama College, Gobo, Wakayama, Japan; e Department of Applied Chemistry and Biochemistry, National Institute of Technology, Wakayama College, Gobo, Wakayama, Japan; University of Maryland School of Medicine

## Abstract

Saccharomyces cerevisiae strain DJJ01 was isolated from Dojoji Temple (Gobo, Wakayama, Japan) for development of local breweries. Here, we report the draft genome sequence of this strain to facilitate comparative genomic studies of yeast strains used for Japanese sake brewing.

## ANNOUNCEMENT

Saccharomyces cerevisiae is used in the fermentation of food (such as Japanese sake) and the production of useful materials (1–3). For example, Kyokai yeast no. 7 (strain K7) is the most extensively used industrial sake yeast strain and is employed in numerous genetic and biochemical studies as a model sake yeast and as a parent strain for breeding (4–8). In this project, S. cerevisiae DJJ01 was isolated from sakura flowers in the precincts of the Dojoji Temple after inoculation to koji medium. Cultivation was carried out at 20°C for 14 days. S. cerevisiae smooth cream colonies could be distinguished morphologically from bacteria and fungi on yeast extract-peptone-dextrose (YPD) plates. PCR results using internal transcribed spacer (ITS) primers (ITS1F, GTAACAAGGTYTCCGT; ITS1R, CGTTCTTCATCGATG) showed good agreement between the amplified sequence of strain DJJ01 and S. cerevisiae. Here, we report the draft genome sequence of S. cerevisiae DJJ01.

Genomic DNA from strain DJJ01 was purified using the ISOIL for bead beating kit (Nippon Gene, Japan) after culturing at 30°C for 24 h in YPD liquid medium. The genomic DNA was further purified using AMPure beads (Beckman Coulter, Inc., USA). Sequencing libraries were prepared using a Nextera XT DNA library preparation kit (Illumina, USA). The library was subjected to short-read sequencing using the MiSeq platform (Illumina) with 250-bp paired-end reads. Low-quality reads were trimmed, and *de novo* assembly was conducted using SPAdes v.3.9.1 (9). Protein-coding genes were predicted using the Microbial Genome Annotation Pipeline v.2.21 (10), local BLAST, and Geneious v.2020.1.2 (Biomatters, New Zealand) (10). Default parameters were used except where otherwise noted.

The assembled genome size was 8,313,543 bp, with 131 contigs (with the size of the largest contig being 365,610 bp), a GC content of 38.3%, an *N*_50_ value of 63,462 bp, and sequence depth of 65.4× from 8,790,476 paired-end Illumina short reads. In total, 262 tRNAs, 86 noncoding regions, 167 assembly gaps, 53 miscellaneous features, and 5,495 protein-coding sequences were annotated.

Genome mapping using short reads from strain DJJ01 against reference sequences of 16 chromosomes of strains K7 (GenBank accession number AP012028) and S288C (GenBank accession number KP263414) was performed to confirm the genome-wide similarity between DJJ01, K7, and S288C. The differences in the nucleotide sequence of the coding sequence region of strain S288C were 3 times larger than those of strain K7. This hints at a potential of this strain for sake production that can be enhanced using molecular breeding technology, eventually expanding the diversity of sake production in the future, and the data presented here may help in brewing Japanese sake to enhance its richness and flavor.

### Data availability.

The draft genome sequence of Saccharomyces cerevisiae strain DJJ01 was deposited under BioProject accession number PRJNA785722 and BioSample accession number SAMN23575053. The sequence of the ITS1 region of strain DJJ01 was deposited in DDBJ/GenBank under accession number LC405963, and genome assembly information was deposited under GenBank accession number JAMCCV000000000.
